# Learning an Embodied Visual Language: Four Imitation Strategies Available to Sign Learners

**DOI:** 10.3389/fpsyg.2018.00811

**Published:** 2018-05-30

**Authors:** Aaron Shield, Richard P. Meier

**Affiliations:** ^1^Speech Pathology and Audiology, Miami University, Oxford, OH, United States; ^2^Linguistics, University of Texas at Austin, Austin, TX, United States

**Keywords:** sign language, Autism Spectrum Disorders (ASD), imitation, language acquisition, visual perspective-taking, American Sign Language (ASL)

## Abstract

The parts of the body that are used to produce and perceive signed languages (the hands, face, and visual system) differ from those used to produce and perceive spoken languages (the vocal tract and auditory system). In this paper we address two factors that have important consequences for sign language acquisition. First, there are three types of lexical signs: one-handed, two-handed symmetrical, and two-handed asymmetrical. Natural variation in hand dominance in the population leads to varied input to children learning sign. Children must learn that signs are not specified for the right or left hand but for dominant and non-dominant. Second, we posit that children have at least four imitation strategies available for imitating signs: anatomical (*Activate the same muscles as the sign model*), which could lead learners to inappropriately use their non-dominant hand; mirroring (*Produce a mirror image of the modeled sign*), which could lead learners to produce lateral movement reversal errors or to use the non-dominant hand; visual matching (*Reproduce what you see from your perspective*), which could lead learners to produce inward–outward movement and palm orientation reversals; and reversing (*Reproduce what the sign model would see from his/her perspective*). This last strategy is the only one that always yields correct phonological forms in signed languages. To test our hypotheses, we turn to evidence from typical and atypical hearing and deaf children as well as from typical adults; the data come from studies of both sign acquisition and gesture imitation. Specifically, we posit that all children initially use a visual matching strategy but typical children switch to a mirroring strategy sometime in the second year of life; typical adults tend to use a mirroring strategy in learning signs and imitating gestures. By contrast, children and adults with autism spectrum disorder (ASD) appear to use the visual matching strategy well into childhood or even adulthood. Finally, we present evidence that sign language exposure changes how adults imitate gestures, switching from a mirroring strategy to the correct reversal strategy. These four strategies for imitation do not exist in speech and as such constitute a unique problem for research in language acquisition.

## Learning an embodied visual language: four imitation strategies available to sign learners

Nearly 60 years of research into the signed languages of the Deaf have unequivocally demonstrated that they are fully comparable to spoken languages in a linguistic and biological sense, utilizing similar brain tissue as spoken languages and organized on the phonological, morphological, semantic, syntactic, and discourse levels (e.g., Klima and Bellugi, 1979; Poizner et al., 1990; Emmorey, 2002; Sandler and Lillo-Martin, 2006). They are acquired naturally by children who are exposed to them and achieve language milestones at similar ages as children acquiring spoken languages (Newport and Meier, 1985), and exist as naturally-occurring, autonomous linguistic systems throughout the world wherever a Deaf community is found. Yet signed and spoken languages are not the same. In recent years, many scholars have investigated the role that modality—the channel through which language is expressed and perceived—plays in linguistic structure, highlighting the ways in which signed and spoken languages may differ (Meier et al., 2002). In this paper we focus on the visual-gestural modality of sign in order to identify a crucial difference between the acquisition of sign and speech. We begin with a discussion of the mental representation of lexical signs as a way to frame the unique challenges entailed in sign acquisition.

**Figure 1:**
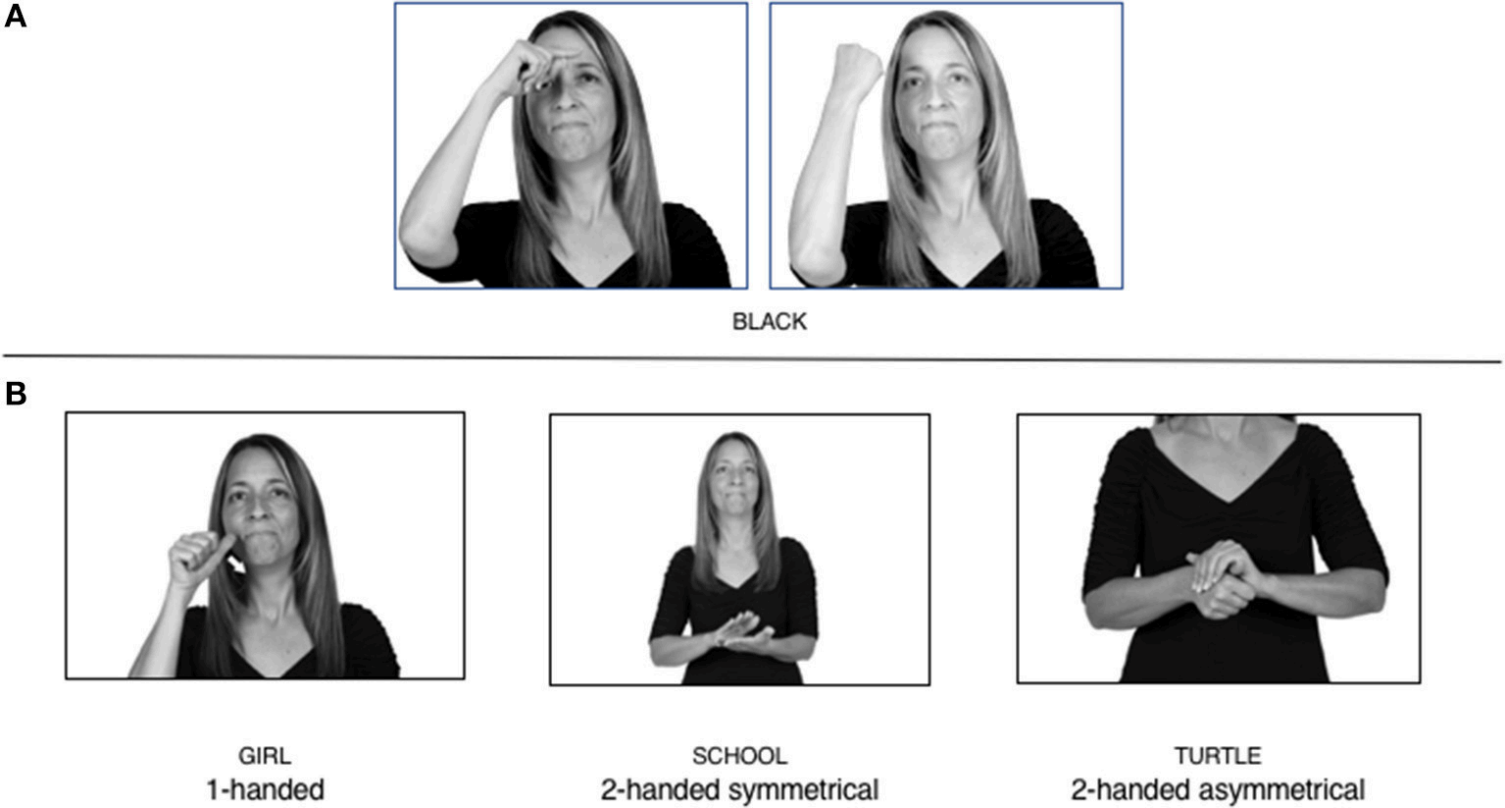
The ASL signs black **(A)** and the signs girl, school, and turtle **(B)**. All photographs, copyright Aaron Shield and Richard P. Meier, are reproduced here and in Table 1 with written permission of the model.

How do we represent signs? We can represent them as they are typically (but not invariably) viewed by an addressee; that is, from a viewpoint opposite the signer. This is the representation most often seen in videos or in linguistics papers, where photos or line drawings show a frontal view, from waist to head, of a signer. The sign black^1^ (Figure 1A) viewed from this perspective, moves to the addressee's left (assuming that the signer is right-handed). Yet movement to the addressee's left is not linguistically significant; if the addressee happens to be seated in the passenger seat beside the signing driver of a (left-hand drive) car, the sign black moves to the addressee's right. The addressee's usual perspective, opposite the signer, is familiar but is not the basis for a linguistically correct description of the sign.

A better linguistic characterization is that the sign black moves to the signer's right, but even this is not quite correct because this description does not capture the way in which left-handed people sign (or even the way in which a right-handed person signs when using the left hand). A still better description is this: in the sign black the active hand moves laterally; the direction of movement is away from the signer's midline (and away from the side of the signer that is ipsilateral to the active hand). To take another example, the sign girl (Figure 1B) makes contact on the signer's cheek, specifically on the cheek that is ipsilateral to the signer's dominant hand. Years of linguistic research have demonstrated that the best description of signs is from the signer's perspective, not the addressee's. Thus, the way in which we generally picture signs does not match the way in which they should be linguistically represented.

Which perspective to take when representing signs has been an issue in attempts to design writing systems for signed languages. In the development of SignWriting, Deaf users instigated a shift from writing signs from the viewer's perspective to the writing of signs from the signer's perspective (Hoffmann-Dilloway, 2017). But, visual representations of signs from the signer's perspective are somewhat unfamiliar; native signers are less accurate and slower to recognize signer-perspective videos of signs than they are to recognize addressee-perspective videos (Emmorey et al., 2009). Maxwell (1980) detected a similar problem in how deaf children decoded drawings of signs in relation to English words (Sign Print). She noted that English print is represented from left to right, but the direction of movement depicted in the drawings of some signs (such as the Signing Exact English plural noun thing-s) was from right to left. As a result, a 48-month-old deaf child misinterpreted the signs as occurring in the reverse order (as s-thing). She also sometimes turned her body so as to share the same orientation as the figure depicted in the book, evidence of the difficulty posed by the illustrations.

These difficulties in correctly representing signs are not just a problem for linguists seeking to understand grammatical descriptions of a signed language or for people interested in representing signs in written form (or for children attempting to read Signing Exact English). They are a fundamental challenge to children and adults who are acquiring a signed language. We posit that these difficulties present problems for acquisition that are unlike the challenges of acquiring speech. The parts of the body that are used to produce and perceive signed languages (the hands, face, and visual system) obviously differ from those used to produce and perceive spoken languages (the vocal tract and auditory system). In this paper we specifically argue that the asymmetric control of the articulators (the dominant and non-dominant hands) that are used to produce signs, the characteristics of the sign language grammar and lexicon, and the multiple strategies available for the imitation of signs have important consequences for language acquisition and processing. We address each of these issues in turn, marshaling evidence from development, second-language acquisition, and atypical learners to support our observations.

### Handedness and the sign lexicon

It is perhaps a trivial statement to note that signed languages are produced with the hands, but a few observations about this fact are in order. First, the articulators are paired; under normal circumstances we have two hands. There is no obvious parallel in spoken languages. There are two lips, but they are not involved in the production of every phoneme. Furthermore, the lips are paired vertically rather than horizontally; the same is true for the top and bottom teeth. We would have to imagine a creature with two mouths in a horizontal configuration, each of which could articulate semi-independently from the other, to obtain an adequate analog.

A second observation is that the hands are controlled semi-independently and show different phonological properties. Signs can be one-handed (as in girl, Figure 1B), two-handed and symmetrical, in which both hands exhibit the same handshape and movement (as in school, Figure 1B), or two-handed and asymmetrical, in which the hands can exhibit different handshapes and in which the non-dominant hand can be static whereas the dominant hand moves (as in turtle, Figure 1B). Battison (1978) discussed the constraints that hold on these three classes of signs in ASL and other signed languages. In the case of one-handed signs, the signer can choose either hand to produce the sign, as signs are not typically specified for left or right^2^. Signers typically employ their dominant hand to produce such signs, though in certain circumstances they may choose to use their non-dominant hand (such as if the dominant hand is holding an object or is otherwise unavailable). The same is true for two-handed asymmetrical signs: the dominant hand acts upon the non-dominant hand, but whether the dominant hand is right or left depends on the individual.

Now let us imagine being a young child who is exposed to a sign language. Handedness is consistently evident by 6 months of age (Butterworth and Hopkins, 1993) or even *in utero* (MacNeilage, 2008), long before children produce their first signs. Let us further imagine that this hypothetical child is an emergent lefty. But all of the adults around him are right-handed, and all he sees is right-dominant signing. This situation must be frequent: it is commonly accepted that about 90% of the general population is right-handed (Corballis, 1980, 1992), and the deaf, signing population shows similar percentages of right-dominance (Conrad, 1979; Bonvillian et al., 1982; Sharma, 2014; Papadatou-Pastou and Sáfár, 2016). How does our imagined child come to understand that he may in fact perform signs with his left hand, when all he sees are examples of right-handed input? Does his strong motor preference for the left dictate his signing, or does a desire to imitate the exact movements of the adults around him motivate him? We cannot know, of course, what the child is thinking, but we can observe whether the child signs with his right or left hand. In this paper we describe several competing imitation strategies that are available to sign learners and hypothesize that different groups of signers may opt for different strategies due to how they interpret the sign imitation/learning task.

Recent work also suggests that handedness plays a role in sign recognition: Watkins and Thompson (2017) found that left- and right-handed adult signers reacted differently to signs produced by left- and right-handed sign models. Left-handed adult signers responded more quickly on a picture-sign matching task when they viewed two-handed asymmetric signs produced by a left-handed model than by a right-handed model, suggesting that the articulatory and perceptual complexity of this sign type is more easily recognized when there is congruency between signer and addressee, perhaps because the addressee can more easily recognize the sign through simulation of the sign through their own motor system. However, Watkins and Thompson also found that for all other sign types (i.e., one-handed signs and two-handed symmetrical signs), both left- and right-handed participants identified signs produced by right-handed models more quickly, suggesting a familiarity effect, since both left- and right-handed signers are exposed to more right-handed signing than left-handed signing. Similarly, Sharma (2014) found that left-handed signers made fewer errors than right-handed signers when forced to produce signs with their non-dominant hand, either because they have more practice viewing and processing signs with non-matched handedness or due to weaker handedness than right-handed signers. There is simply no analog to this situation in speech: there is no anatomical component of the vocal tract that varies in such a significant way in a subgroup of the population and that could have such important effects on both production and comprehension of language as does hand preference in sign^3^.

### Perspective-taking

**Figure 2:**
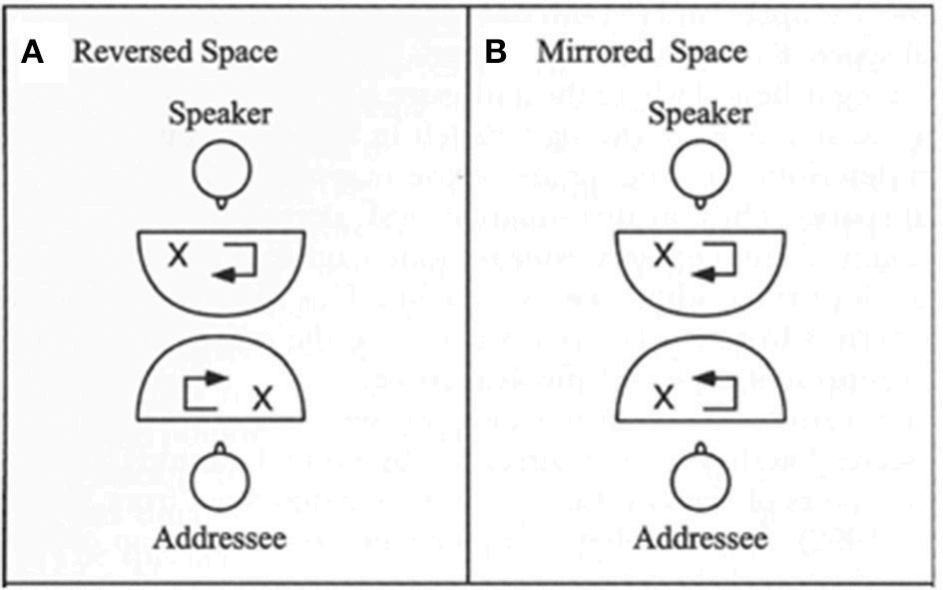
A signed spatial mapping correctly reversed **(A)** and incorrectly mirrored **(B)** (reproduced from Emmorey (2002: 415)). Used by permission. All rights reserved.

Like hand preference, the role of visual perspective-taking in sign learning represents a unique challenge for sign learners. Many scholars have noted the role that the three-dimensional signing space plays in sign grammar: the physical space in front of the signer is exploited for pronominal and anaphoric reference, for verb agreement, and for the description of spatial arrays (Bellugi et al., 1990). As far as we know, signed languages universally depict such constructions from the perspective of the signer. Courtin and Melot (1998: 85) point out that such constructions require “a visual perspective change; the addressee has to reorient the linguistic space according to the angle existing between himself and the signer.” Particularly difficult are descriptions of spatial arrays (that is, signed depictions of spatial configurations or movements), as discussed by Emmorey et al. (1998, 2000). Emmorey (2002) provides a schematic for how such constructions are produced and understood. In Figure 2, the arrow indicates the direction of movement of a referent, which is represented by the X and is first located in the sign space in front of the signer. The signer wishes to communicate that the referent moved through space, first to the left, then forward, and finally back to the right. The direction of movement is only properly repeated if reversed by the addressee (as in Figure 2A), whereas mirroring the movement (as in Figure 2B) suggests an incorrect interpretation (right, forward, left). Bellugi et al. (1990: 287) note the challenges that such structures pose to learners: “The young deaf child is faced with the dual task in sign language of spatial perception, memory, and spatial transformations on the one hand, and processing grammatical structure on the other, all in one and the same visual event.” Unsurprisingly, linguistic structures in sign that crucially depend on such mental transformations appear later in development than might otherwise be expected (Lillo-Martin et al., 1985; Newport and Meier, 1985), since “the young deaf child, unlike his or her hearing counterpart, must acquire non-language spatial capacities that serve as prerequisites to the linguistic use of space” (Bellugi et al., 1990: 287).

Shield (2010) proposed that visual perspective-taking and spatial transformations are necessary not only for comprehending complex descriptions of spatial arrays but also for acquiring the phonological form of individual lexical signs. Lexical signs are acquired much earlier in development than are complex spatial descriptions. Unlike the spatial descriptions described by Emmorey (2002), lexical signs are not (typically) specified for right or left, but for the dominant and non-dominant hand. This difference in the use of space has important consequences for the sign-learning child, who must realize that the use of space in lexical signs is fixed and does not make reference to space itself, whereas the descriptions of spatial arrays discussed by Emmorey are linguistic devices for talking about space.

**Figure 3:**
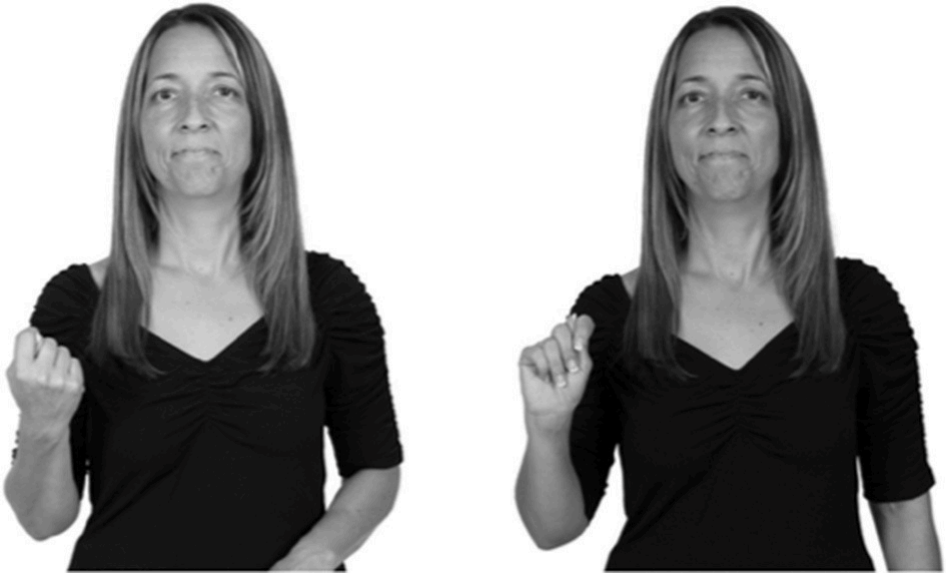
The ASL signs tuesday **(left)** and bathroom **(right)**. Photographs are reproduced with written permission of the model.

Shield (2010) suggested that some signs engage perspective-taking skills in more challenging ways than others, and that certain types of learners would produce specific error types in the process of learning and reproducing these signs, especially very young typically-developing children as well as individuals with autism spectrum disorder (ASD), who may have difficulties with visual perspective-taking (Hamilton et al., 2009). With regard to the sign types that may be challenging, Shield hypothesized that lexical signs exhibiting *lateral path movements* (from the ipsilateral side of the body to the contralateral side of the body or vice versa) as well as *inward–outward movements* (movements originating at a point distal from the signer's body and moving to a point more proximal to the signer's body or vice versa) require learners to engage perspective-taking skills in order to form correct phonological representations of signs in ways that other types of path movement (such as vertical movements in an upward or downward direction) do not. Likewise, he argued that *inward–outward palm orientations*, such as those found in the ASL signs tuesday and bathroom (Figure 3) could also engage perspective-taking, because these palm orientation values appear differently from the signer's and viewer's perspectives.

### Imitation

Like children acquiring speech, children learning sign must imitate the linguistic symbols produced by the language models around them^4^. However, unlike the learning of spoken words, we hypothesize that there are multiple strategies for the imitation of lexical signs, and that not all of these strategies will result in a correctly-formed sign. The psychological literature has distinguished two kinds of imitation strategies: *anatomical* imitation and *mirror* imitation (Koski, 2003; Franz et al., 2007; Press et al., 2009). In anatomical imitation the imitator activates the same muscles as the model being imitated, such that, for example, he raises his right arm to imitate the model's raised right arm, or his left arm to imitate the model's raised left arm. In mirror (or *specular*) imitation, the imitator performs the action as if looking in a mirror (e.g., raising his left arm to mimic the model's lifted right arm). Pierpaoli et al. (2014) found that adults tend to spontaneously engage a mirror imitation strategy more often than an anatomical strategy unless given specific instructions about which limb to use, suggesting that the mirror strategy is a default imitation strategy for typical adults.

For sign-learning children, however, neither the anatomical strategy nor the mirror strategy is correct, for two reasons. First, the anatomical strategy is inappropriate for learners imitating a model with different hand dominance: signs are not specified for the right or left hand but for dominant and non-dominant. Thus the anatomical strategy will fail when the signer and the learner are discordant in hand dominance.

Second, though some signs can be mirrored without error (e.g., signs exhibiting inward–outward movements and palm orientations as in Figure 3 above), the mirroring of signs containing lateral movements (as in the sign black, Figure 1A) will lead to movement reversal errors if signer and learner are both right-handed or both left-handed. In this case, only a *reversing* strategy will result in the production of the correct form. We follow Emmorey (2002) in using the term “reversing” since it implies that the imitator must perform a mental spatial transformation of what he or she sees in order to produce the correct form. To this strategy, we add the caveat that learners must monitor the handedness of the signer and compare it to their own; if hand dominance is discordant, learners may correctly deploy the mirroring strategy.

In addition to these three strategies, yet another imitation strategy is available to learners. Learners may reproduce what they see from their own perspective. This is a *visual matching* strategy because the child's imitative movements match the appearance of what she sees. Let us imagine a child who has adopted this strategy and who is facing the signer. The child sees a sign which originates at a point distal from the signer and which moves toward the signer's own body. The child could interpret the signer's movement in an absolute sense. She could then reproduce the sign as beginning relatively proximal to her own body and ending at a point distal from her body. Similarly, she could imitate signs exhibiting outward-facing palm orientations (as in bathroom, Figure 3) with her palm facing inward toward her own body, thus reversing the palm orientation parameter. Thus, the visual matching strategy would lead to movement and palm orientation errors on signs exhibiting inward–outward movements and palm orientations. Note that this strategy yields predictions about inward–outward movements and palm orientations, but not about hand selection.

To summarize, it appears that children learning sign have at least four possibilities for imitating signs during acquisition:

1. Anatomical strategy: Activate the same muscles as the model, regardless of the hand dominance of the signer.
2. Mirroring strategy: Produce a mirror image of what the signer does.
3. Visual matching strategy: Reproduce the sign as it appears from the learner's perspective.
4. Reversing strategy: Perform a mental spatial transformation on the observed sign and reproduce what the signer does after checking for differences in hand dominance.

Table 1 summarizes each imitation strategy, the conditions under which each strategy will fail, the types of lexical signs that could be susceptible to error when employing each strategy, and the error types that are predicted.

**Table 1:** Four imitation strategies in sign learning and predicted error types.

**Strategy**	**Under what circumstances will this strategy lead to errors in sign formation?**	**Predicted errors**	**Example signs**
**Anatomical:** Activate the same muscles as the model	When imitating one-handed and two-handed asymmetrical signs if handedness of signer and learner is discordant	Hand switches on one-handed signs such as bathroom and two-handed asymmetrical signs such as tomato	Sign: bathroom (produced by a left-handed signer)
			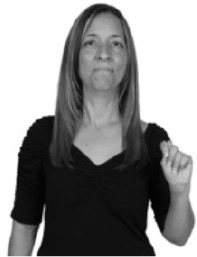
			Produced (by a right-handed signer) as:
			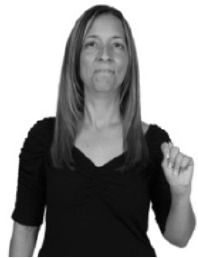
**Mirroring:** Produce a mirror image of what the signer does	When imitating signs exhibiting lateral movements if handedness of signer and learner is concordant; for one-handed signs if handedness of signer and learner is concordant	Lateral movement reversals (on signs such as black, summer, because, farm, ugly, dry, we, committee, congress, board, senate, atlanta, toronto, and poland); hand switches on one-handed signs such as bathroom	Sign: black
			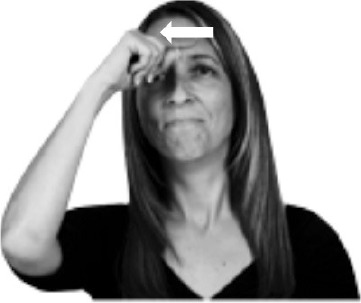
			Produced as:
			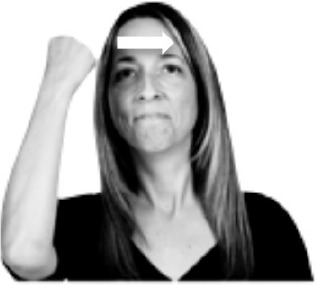
			Sign: bathroom
			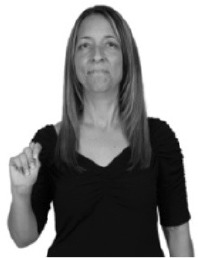
			Produced (by a right-handed signer) as:
			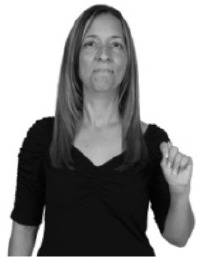
**Visual matching:** Reproduce the sign as it appears from the learner's perspective	When imitating signs exhibiting inward–outward movements and palm orientations; when imitating signs exhibiting lateral movements if handedness of signer and learner is concordant	Inward–outward palm orientation reversals on signs such as tuesday and bathroom; inward–outward movement reversals on signs such as want; lateral movement reversals	Sign: bathroom
			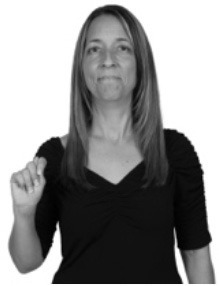
			Produced as:
			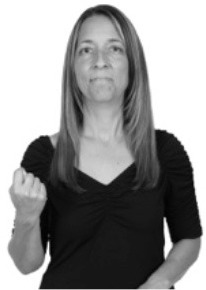
**Reversing:** Reproduce what the signer does after performing a mental spatial transformation and checking for handedness^5^	Never	None	Sign: bathroom
			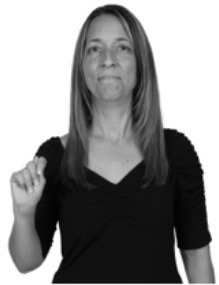
			Produced as:
			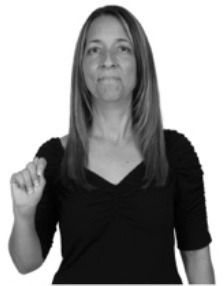
			or, for left-handed signers:
			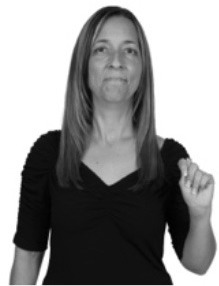

Which strategy or strategies do sign learners adopt, and how would we know? We predict that the difficulties posed by the interaction of sign type (one-handed, two-handed symmetrical, and two-handed asymmetrical), natural variation in handedness, and the four imitation strategies available to learners will lead some sign learners to make specific types of errors, namely *hand-switches, lateral and inward–outward movement reversal errors*, and *inward–outward palm orientation reversal errors*, depending on the type of strategy or strategies adopted. We first turn to evidence from published studies on gesture imitation and sign acquisition by typical and atypical hearing and deaf children, as well as by typical adult learners. We then present two new studies of gesture imitation by non-signers, sign learners, and fluent signers to show how exposure to a sign language changes how adults approach imitation. Throughout we demonstrate that hearing and deaf, typical and atypical, children and adults produce errors that reveal the specific difficulties presented by learning a visual language.

## Studies of gesture imitation and sign acquisition by typical hearing and deaf children

Children begin imitating the actions and gestures of the people around them early in development, for example producing early communicative pointing gestures and conventional gestures such as the “wave bye-bye” gesture by 12 months (Bates, 1979; Carpenter et al., 1998). Studies of the ways that typical infants imitate others suggest that they may shift from an initial visual matching strategy to a mirroring strategy in the second year of life. Evidence for this hypothesis comes from studies on infants' ability to perform role reversal imitation (Tomasello, 1999; Carpenter et al., 2005), that is, performing an action toward another person in the same way that the action was performed on the child. Two kinds of role reversal imitation have been identified: self-self role reversal, in which the child performs an action on his own body in imitation of an action that the adult performed on her own body (e.g., the infant pats his own head after the adult pats her own head), and other-other role reversal, in which the child performs an action on the adult's body in imitation of an action that the adult performed on the child's body (e.g., the infant pats the adult's head after the adult pats the infant's head). Carpenter et al. (2005) found that 50% of their sample of typical 12-month-old infants and 90% of their sample of typical 18-month-olds performed self-self role reversals, suggesting that this ability develops and strengthens during the second year. The ability to perform such reversals could be key for the development of the reversing or mirroring strategies of imitation, as children imitate not just what they *see* but what others *do*. By the time typical children are preschool age, they are able to imitate the actions of others with high fidelity (Ohta, 1987), and no longer appear to engage the visual matching strategy of imitation.

How does the child's ability to imitate action contribute to the acquisition of signs? Movement errors have frequently been reported for young, typical deaf children acquiring sign (Siedlecki and Bonvillian, 1993; Marentette and Mayberry, 2000; Meier, 2006; Morgan et al., 2007). A problem in interpreting this literature, which is largely based on the observation of naturalistic data, is that it can be difficult to separate errors that arise due to young children's immature motor control from errors that arise due to the perceptual challenges that are the subject of this paper. Crucially, there are very few reports of the development of palm orientation. Palm orientation is the parameter that could shed the most light on the issues raised in this paper because data on inward–outward palm orientations can tell us if children have adopted a visual matching strategy (in which they are likely to reverse inward–outward palm orientations) or have acquired either a reversing or a mirroring strategy (both of which would result in the correct imitation of palm orientation).

To address this question, Shield and Meier (2012) examined 659 tokens in the database of children's early sign productions of four typical deaf children between 9 and 17 months of age (on which Cheek et al., 2001, had based a previous report). This examination revealed 14 tokens (6 inward substitutions and 8 outward substitutions) of reversed inward–outward palm orientation in a database of 659 signs produced by typical deaf children in the first year and a half of life. Thus, it appears that very young typical deaf children do sometimes reverse the palm orientation parameter in a way that appears consistent with their use of the visual matching imitation strategy. However, it is unclear if they do so systematically. We do not yet have a systematic, longitudinal examination of children's acquisition of those sign types that are directly relevant to the hypotheses presented above.

One feature of the way in which infants are socialized to language may contribute to their reconciliation of the different appearances that an individual sign has when viewed from different perspectives. Infants are sometimes seated opposite their parent—say, when they are in a high chair being fed. But infants may also be seated on the parent's lap; in this instance their perspective on the world is closely aligned with that of the parent. Several studies of child-directed signing by Deaf caregivers have shed light on these interactions. Maestas y Moores (1980) studied how American Deaf parents interacted with their infants (*n* = 7); the infants ranged in age from less than a month to 16 months. She found that Deaf parents commonly signed in front of the infant while the infant was seated on the parent's lap such that the viewpoint of parent and infant were shared. Parents also commonly signed on the infants' bodies, molded their hand configurations, and guided their hand movements, thus providing kinesthetic as well as visual feedback to their children. Similar results have been found for Deaf British mothers who use British Sign Language (Woll et al., 1988).

In a later study, Holzrichter and Meier (2000) reported that four Deaf mothers of deaf children between 8 and 12 months of age displaced signs with a place of articulation on the face onto their children's bodies about 18% of the time (21 of 116 tokens); these instances occurred when there was no eye contact between parent and child during or before the articulation of the sign, such as when the child was sitting on the mother's lap facing away from her. Pizer et al. (2011) describe an interesting example of such an interaction between a Deaf mother and her 18-month-old deaf child. The child was seated on her mother's lap; the mother labeled the colors of the blocks that were on the floor in front of them. The mother produced the ASL signs green, blue, and yellow in the neutral space in front of the two of them. She then produced the sign orange on her child's mouth rather than on her own body, as normal signing would dictate. Why did the mother do this in this instance? If she had articulated the sign in contact with her own mouth, the sign would not have been visible to the child (because the mother was behind her daughter). When, in these instances, the mother signed green, blue, and yellow in front of the child and orange on the child's mouth, she enabled her child to witness these signs from the signer's own perspective, rather than from the more typical addressee perspective.

## Studies of gesture imitation and sign acquisition by hearing and deaf children with ASD

We also find indications of the challenges presented by learning sign in studies of atypical learners. Children with ASD, both hearing and deaf, show distinctive patterns in imitation.

### Hearing children with ASD

Though language impairment is not considered a core feature of ASD (American Psychiatric Association, 2013), many children with ASD exhibit abnormal language in both speech and sign. A significant minority of children with ASD are considered minimally-verbal (Tager-Flusberg and Kasari, 2013), with expressive vocabularies under 50 words. Manual signs have long been used as an alternative communication strategy for such children, with varying degrees of success (Carr, 1979; Bonvillian et al., 1981, for reviews). In general, minimally-verbal hearing children with ASD are not exposed to, and do not learn, a fully-fledged sign language such as ASL with its syntax and morphology, but instead see a restricted set of lexical signs that are used to communicate basic wants and needs, akin to Baby Signs (Acredolo and Goodwyn, 2002). The published reports on hearing children with ASD who are exposed to signs are unfortunately not useful for the purpose of testing our hypotheses, although Bonvillian et al. (2001) speculate that an unexpectedly high preference for left-handed signing in their subjects may be attributable to mirroring. We now turn to the literature on gesture imitation by children with ASD.

Various studies have observed that hearing children with ASD do more poorly in general on gesture imitation tasks than typical children, and numerous hypotheses have been advanced to account for these deficits. Edwards (2014) recently performed a meta-analysis of 53 studies on imitation in ASD. She found that individuals with ASD performed on average about 0.8 standard deviations below non-ASD individuals on the imitation tasks contained in the studies, despite important differences between the individual studies depending on the nature of the task and the characteristics of the subject samples. In the section that follows we do not claim to account for all children with ASD, but rather focus on a subset of studies that describe a unique pattern that thus far has only been documented in the imitative behavior of children with ASD.

At least four studies have shown that children with ASD, unlike typical children, produce gesture imitations suggestive of the visual matching imitation strategy. Ohta (1987) was the first to report such errors (which he called “partial imitations”): three of 16 children with ASD between the ages of 6;3 and 14;4 (mean age 10;2) imitated a “wave” gesture (in which the experimenter's open palm was oriented toward the child) with their palms facing inward toward themselves, consistent with the visual matching imitation strategy. Crucially, no member of an age- and IQ-matched control group or of a second control group of 189 typical preschoolers ages 3–6 imitated the wave gesture in this way, suggesting that this imitation strategy does not occur in typical development beyond a very early age.

Other studies have replicated this striking finding. Smith (1998) found that hearing children with ASD made significantly more 180-degree reversal errors (e.g., palm toward the viewer rather than away from him) than age-matched language-impaired and typically developing children when imitating ASL handshapes and bimanual gestures. Whiten and Brown (1998: 270–271) also found that hearing children with ASD made similar gesture imitation errors, highlighting

responses in which the imitating subject creates an *action which to him will look similar to what he saw when he watched the demonstrator, instead of what the demonstrator would see*. He fails to translate appropriately, or “invert” the action to his own perspective as actor. An example is “peekaboo,” performed by the demonstrator with palms toward her own face, and sometimes inaccurately imitated such that the palms are oriented away from the imitator's face (i.e., the actor sees the backs of the hands both when the demonstrator performs the act, and when he himself attempts it) (*emphasis ours*).

Adding to these findings, Hobson and Lee (1999) provide a crucial link between the reversal errors in gesture imitation and the role reversal skills described by Tomasello (1999). They found that adolescents with ASD were significantly less likely to imitate a self-oriented action (wiping their own brow with a toy frog after an adult did so) than were age- and language-matched intellectually-disabled children: only five of 16 children with ASD performed the self-oriented action while 14 of 16 of the control children did so. This finding suggests that it is indeed this early development of role reversal skills that enables typical children to transcend the visual matching strategy. That visual matching strategy has now surfaced in multiple studies of how children with ASD imitate gestures.

### Deaf children with ASD

More recently, Shield and colleagues have published a number of studies describing the acquisition of ASL by deaf children with ASD who have Deaf parents (Shield and Meier, 2012; Shield, 2014; Shield et al., 2015, 2016, 2017a,b; Bhat et al., 2016). The first report (Shield and Meier, 2012) described the formational errors produced by five native-signing children with ASD (four deaf children and one hearing child of Deaf adults) ranging in age from 4;6 to 7;5. These children were compared to a control group of 12 typical native-signing deaf children between the ages of 3;7 and 6;9. The data came from spontaneous signing produced under naturalistic conditions and from a fingerspelling task (in which children were asked to spell English written words with their hands). Despite lifelong exposure to ASL, three of the children with ASD (ages 5;8, 6;6, and 7;5) reversed the palm orientation of 72 of 179 (40.2%) fingerspelled letters such that the children's palm faced toward their own body rather than outward. None of the 12 typical deaf children produced any such palm orientation reversals. These reversals are consistent with the visual matching strategy of imitation and are nearly identical to the errors produced by hearing, non-signing children with ASD in the previously-discussed studies of gesture imitation (Ohta, 1987; Smith, 1998; Whiten and Brown, 1998). The three children with ASD who made such errors had lower parent-reported language scores (*M* = 36.67, *SD* = 13.61, range 26–52) on the Language Proficiency Profile-2 (LPP-2; Bebko et al., 2003) than those children who did not make such errors, including the 12 typical deaf children (*M* = 90.25, *SD* = 17.07, range 59–112) or the child with ASD who did not make any palm reversals (= 90). This difference was significant [*t*_(14)_ = 5.23, *p* < 0.001], suggesting that children with lower receptive and expressive language skills may be more prone to making such errors.

If the palm orientation reversals exhibited by native-signing children with ASD are the result of the visual matching imitation strategy, then how do such children perform on gesture imitation tasks? Two studies have shown that even deaf children who are exposed natively to a sign language nonetheless show difficulties with gesture imitation. In his unpublished dissertation, Shield (2010) asked 12 typical deaf children and 17 deaf children with ASD to imitate nonsense signs similar to ASL signs. He divided up the target stimuli into test items (hypothesized to require a reversing strategy in order to be imitated correctly, i.e., with lateral path movements) and control items (which do not require a reversing strategy in order to be imitated correctly, i.e., with up–down path movements). The children with ASD made significantly more imitation errors than typical controls overall, as well as significantly more errors on test items than control items, suggesting that gestures that require a reversing imitation strategy can be particularly difficult for such learners. The children with ASD also had significantly lower language scores on the LPP-2 (*M* = 66.25, *SD* = 31.49) than the typical children (*M* = 90.25, *SD* = 17.07), again indicating a relationship between these errors and overall language abilities. Children with ASD made significantly more errors on inward–outward palm orientations than on any of the other item types or parameters, which may be a sign of the visual matching imitation strategy. Thus, the observation of these palm orientation reversals in gesture imitation by deaf, signing children provides a link between the reversed signs observed by Shield and Meier (2012) in spontaneous and elicited production of ASL and the reversed gestures observed in hearing children with ASD by Ohta (1987), Smith (1998) and Whiten and Brown (1998). All of the errors indicate that some children with ASD use a visual matching strategy in imitation far beyond the age that typical children stop doing so.

Shield (2010) also examined whether right-handed children switched hands during the task as a way of avoiding the reversing strategy when imitating the right-handed investigator (thereby using the mirroring strategy instead). Both typical and ASD children switched hands significantly more often on test items than control items, suggesting that both groups preferred to avoid the reversing strategy for gestures that were more difficult to imitate. Moreover, younger children switched hands more often than older children, which implies that exposure to and practice with imitation of gestures renders these processes easier over time.

More recently, Shield et al. (2017b) examined the ability of 14 deaf children with ASD between 5 and 14 years old (*M* = 9.5) and 16 age- and IQ-matched typical deaf children to imitate a series of 24 one-handed gestures exhibiting inward–outward movements and palm orientations and up–down movements and palm orientations. They found that children with ASD made significantly more palm orientation errors than typical children (though movement direction errors were largely absent in both groups). Both groups were also inconsistent in the hand that they used to imitate the gestures, possibly to avoid the reversing strategy: on average children with ASD switched hands in 5.67 of 24 trials (23.6%), while typical children switched hands in 3.26 of 24 trials (13.6%). However, note that 10 of 16 typical deaf children and 7 of 14 deaf children with ASD were consistent in using the same hand to imitate all of the trials; these children never switched hands.

Taken together, these studies lead us to think that the imitation of certain types of signs and gestures is particularly difficult for hearing and deaf children with ASD. The inward–outward palm orientation reversal errors identified in studies of gesture imitation by hearing and deaf children with ASD (Ohta, 1987; Smith, 1998; Whiten and Brown, 1998; Shield, 2010; Shield et al., 2017b) and in the sign language of some hearing and deaf children with ASD (Shield and Meier, 2012) suggest that some children with ASD employ the visual matching strategy in gesture imitation, and that this approach to imitation can then influence how children produce signs on their own. Typical children do not appear to employ this strategy once they have mastered role reversal during the very earliest stages of language development. Both typical children and children with ASD switch hands when imitating gestures hypothesized to require the reversing strategy in order to be imitated correctly, thus resorting to the less-difficult mirroring strategy.

## Studies of gesture imitation and sign acquisition by typical adults

In this section, we add to the evidence from studies of children, by reviewing several studies of how typical adults learn signs and imitate gestures. We ask if adults who are learning a sign language exhibit patterns like those described for children, and we ask how adults who have no exposure to sign imitate gestures.

### Sign learning

Rosen (2004) studied 21 adult beginning learners of ASL in a 15-week course and described the types of errors they made in articulating signs. He predicted error types based on perceptual and articulatory factors; here we discuss only the former. He noted that perceptual errors would be rooted in “the physical stance from which the learner views the input source such as the teacher” and would occur “when signers either mirror or make parallel their signs with those of the teacher” (p. 38). Such perceptual errors could then lead to a situation wherein “signers may reverse the handshape, location of contacts, direction of movements, and the orientation of palms within lexical signs as compared to their teacher” (p. 38). As he predicted, Rosen found that adult learners of sign made location, movement, and palm orientation errors based on what he called “mirrorization” and “parallelization.” In our terminology, “mirrorization” errors reflect either the mirroring or anatomical strategy and “parallelization” errors reflect the visual matching strategy. Mirroring errors included reversals of lateral movements; anatomical errors were evidenced by hand switches from dominant to non-dominant. Visual matching errors included palm orientation reversal errors such as the ASL sign door produced with palms facing inward rather than outward. Thus, Rosen found that adult learners of sign struggled with particular types of signs and utilized, in our terms, the mirroring, anatomical, and visual matching strategies to produce them. Unfortunately, he included no quantitative analyses so we do not know how frequently the beginning learners made such errors. Nonetheless, the documentation of these error types in the literature is helpful insofar as it suggests that some signs are more difficult to learn than others, and that typical adults employ several of the imitation strategies we describe in this paper.

### Gesture imitation

We again look to studies of gesture imitation to verify if the errors observed in sign production could be the result of imitation processes. Shield (2010) asked 24 hearing, right-handed undergraduate students who were naive to sign to imitate 48 manual gestures, half of which were extant ASL signs and half of which were nonsense gestures created by modifying the ASL signs. By hypothesis, half of the gestures required a reversing strategy in order to be imitated correctly (i.e., lateral and inward–outward path movements and inward–outward palm orientations) and half did not (i.e., up–down path movements and palm orientations).

The undergraduates made significantly more errors when imitating ASL signs and nonsense gestures hypothesized to require the reversing strategy in imitation (e.g., exhibiting a lateral path movement) than on control items. Signs involving a lateral movement were particularly vulnerable to error: 23 of 44 tokens (52.3%) of the sign black (which moves from the contralateral side of the forehead to the ipsilateral side; see Table 1) contained a movement reversal error, while 16 of 44 tokens (36.4%) of the sign flower, which also entails a lateral path movement across the face, contained a movement reversal error. Two of the subjects imitated all gestures with their left hand (despite being right-handed), thus employing the mirroring strategy and avoiding the reversing strategy. Unlike children with ASD, however, the undergraduates had no difficulty with inward–outward movements or palm orientations and did not appear to use the visual matching strategy.

Thus, this study suggests that typical adults tend to engage a mirroring strategy when imitating novel gestures, which is successful except in the case of lateral path movements when handedness is shared between model and subject. In such cases, typical adults made lateral movement reversal errors or switched hands in order to mirror the gesture correctly.

## Does sign language exposure change how learners imitate?

We have shown that the mirroring and visual matching strategies both lead to specific kinds of imitation errors; furthermore it appears that typical and atypical children as well as typical adults produce errors consistent with these strategies in their signing and gesture imitation. We now present two new studies to further examine our hypotheses. We ask if sign language exposure can change how learners imitate gestures. Specifically, we hypothesize that sign language exposure could shift typical learners from a mirroring strategy to a reversing strategy due to practice with reversing.

### Study 1: mirroring and signer experience

#### Methods

To test the hypothesis that sign exposure may enable typical adult learners to shift from a mirroring strategy to a reversing strategy, we recruited non-signers, sign learners (intermediate ASL students), and fluent signers for a study of gesture imitation.

##### Stimuli

We created 48 gesture stimuli based on four palm orientations (up, down, in, out), six movements (inward toward the body, outward from the body, up, down, ipsilateral→contralateral, contralateral→ipsilateral), and two handshapes (the 1- and 5-handshapes); see Table 2. Each palm orientation type was combined with each movement type to create 24 base gestures; each of these gestures was then filmed twice, once with a 1-handshape (with the index finger extended and all other fingers retracted) and again with the 5-handshape (with all fingers extended). Each videotaped stimulus lasted 1.5 s. None of the gestures were extant ASL signs; thus, they were meaningless for signers and non-signers alike.

**Table 2:** Gesture stimulus types by palm orientation and direction of movement.

			**Palm orientation**			
			**Horizontal**		**Vertical**	
**MOVEMENT**			**Out**	**In**	**Up**	**Down**
	**Vertical**	**Up**	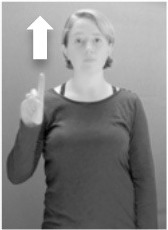	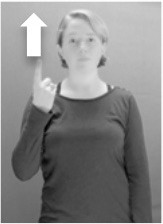	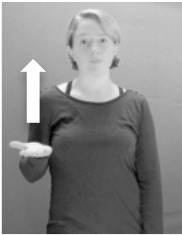	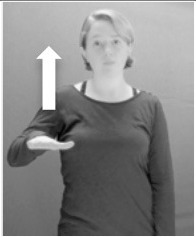
		**Down**	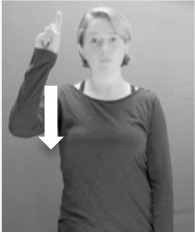	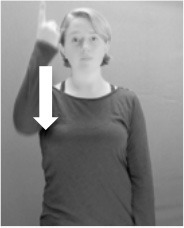	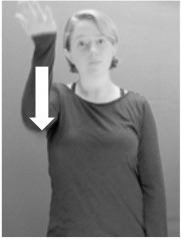	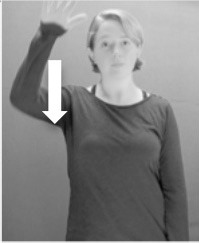
	**Horizontal**	**In**	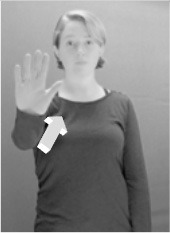	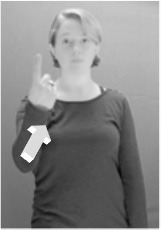	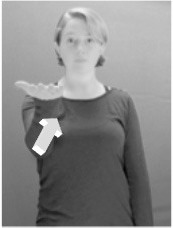	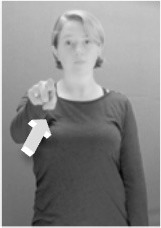
		**Out**	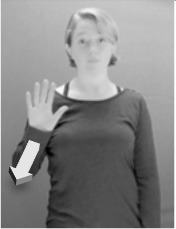	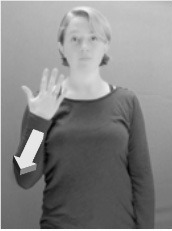	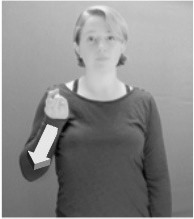	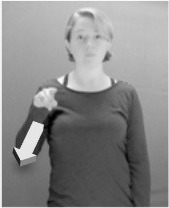
	**Lateral**	**Ipsi-Contra**	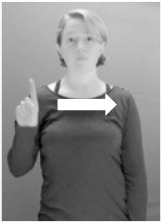	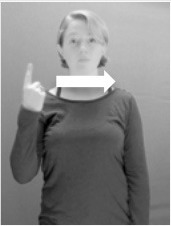	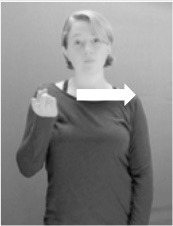	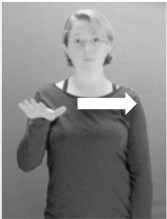
		**Contra-Ipsi**	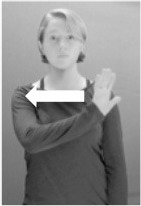	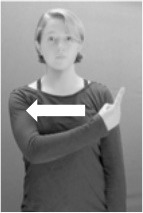	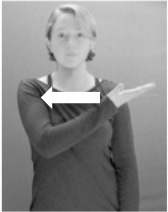	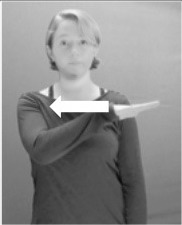

*Every gesture type above was shown to participants twice, once with a 1-handshape and once with a 5-handshape*.

*^a^ Example of a stimulus with a 1-handshape (index finger extended)*.

*^b^ Example of a stimulus with a 5-handshape (all five fingers extended)*.

We hypothesized that all subjects would be able to imitate gestures with vertical and horizontal movements since these can be imitated using the mirroring strategy. However, we predicted that subjects with exposure to ASL would imitate gestures with lateral movements more accurately than non-signers, since these must be imitated using the reversing strategy. We did not predict that any of the groups would have difficulty with the four palm orientations, since these can also be imitated using a mirroring strategy. In order to ensure that all participants would have the opportunity to engage the reversing strategy, we verified the handedness of each participant and then used either a right- or left-handed version of the stimuli, such that every participant imitated a model with concordant handedness. The left-handed version of the stimuli was made by flipping the right-handed stimuli horizontally; thus, the stimuli presented to left- and right-handed participants were identical in every aspect, save for the apparent handedness of the model.

##### Procedure

Participants stood in front of a 17″ MacBook laptop computer, which was placed approximately at eye level three feet away. Participants were instructed to reproduce each gesture as accurately as possible. Each participant viewed each of the 48 gesture stimuli in one of two pre-established random orders; no stimuli were repeated. A 3-s pause followed each gesture stimulus during which participants were asked to imitate the gesture observed.

##### Participants

We recruited three groups of participants: (1) non-signing undergraduate students at Boston University who had never had any exposure to sign language (*N* = 34; all right-dominant, 19 females), (2) sign learners, students who were then enrolled in the fourth or fifth semester of an ASL course (*N* = 25; 23 right-dominant, 22 females), and (3) fluent signers, either professional sign language interpreters or Deaf adults (*N* = 18; all right-dominant, 12 females)^6^.

##### Coding

Each trial was coded blindly by a Deaf native signer for movement direction and palm orientation values so that the coder did not know what the stimulus gesture had been. There were two values per stimulus, a movement value and a palm orientation value. A second coder, a fluent signer, then matched the coded trials to the target movement and palm orientation values and re-coded each trial as correct or incorrect. Any movement or palm orientation value other than the target was considered an error. In order to assess intercoder reliability, a third coder (also a fluent signer) re-coded 20% of the trials. There were 10 disagreements out of 288 re-coded trials; Cohen's κ was 0.97 for palm orientation (6 disagreements out of 288 trials) and 0.98 for movement (4 disagreements out of 288 trials), indicating very high levels of agreement.

##### Statistical analysis

We fit a generalized linear mixed-effects model using error frequency as the dependent variable. The independent variables were experience (non-signer, sign learner, or fluent signer) and gesture type (vertical, horizontal, or lateral movements; up–down or in-out palm orientation). The mixed effects were necessary to model the repeated measures design of the gesture type variable.

#### Results

Non-signers erred on 6.85% of the 48 gestures imitated (*M* = 6.56 errors, *SD* = 4.62), sign learners erred on 2.63% of gestures imitated (*M* = 2.52 errors, *SD* = 2.29), and fluent signers erred on 1.39% of gestures imitated (*M* = 1.33 errors, *SD* = 1.88). Experience was a significant predictor of performance, *X*^2^_(2)_ = 36.03, *p* < 0.0001. *Post-hoc* Tukey comparisons found that non-signers produced significantly more errors than either sign learners (*z* = 4.30, *p* < 0.001) or fluent signers, (*z* = 5.59, *p* < 0.001). The difference between the sign learners and the fluent signers was not quite significant (*z* = 2.08, *p* = 0.09).

##### Movement items

**Figure 4:**
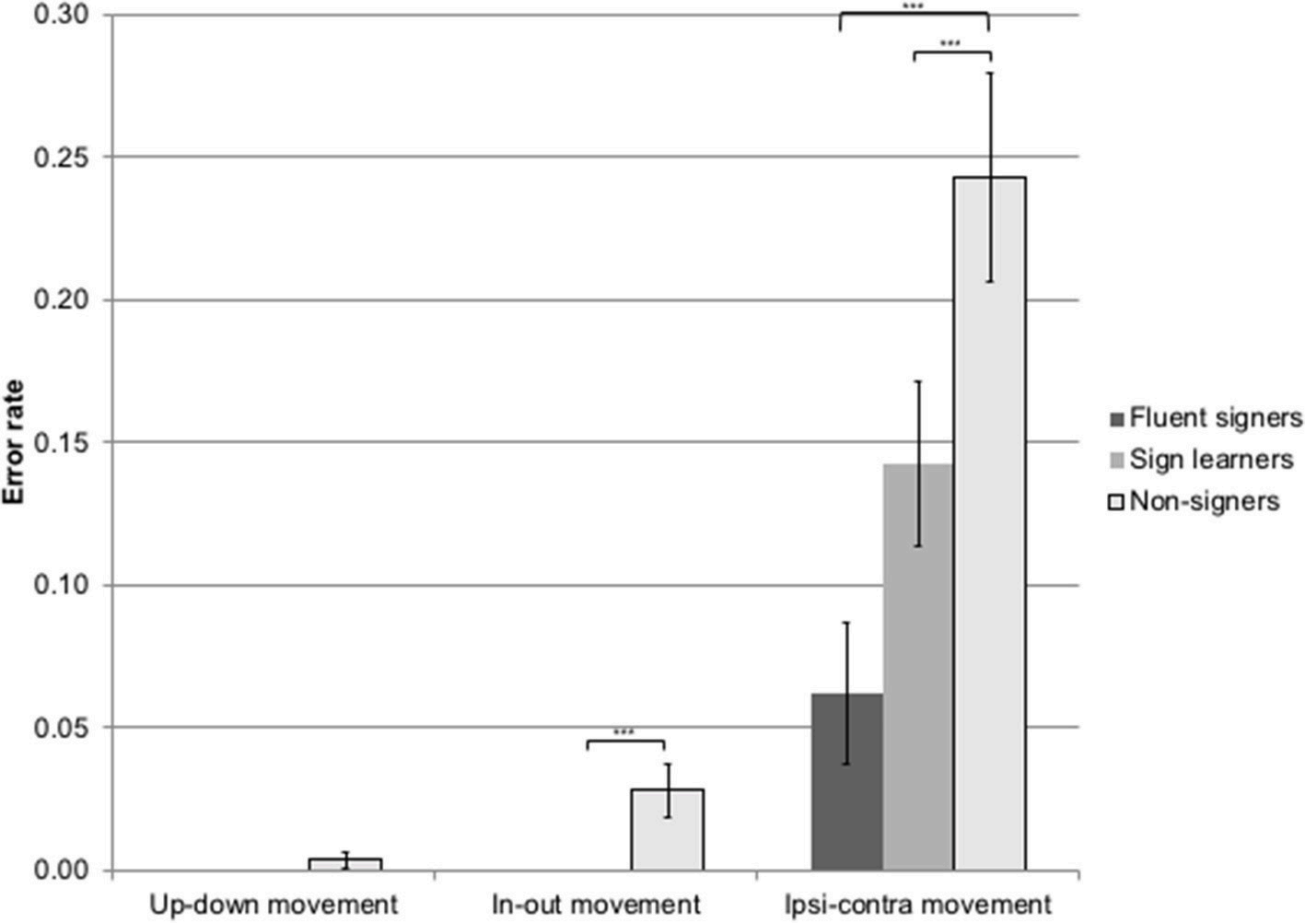
Error rate by movement type for non-signers, sign learners, and fluent signers. ^***^p < 0.001.

Non-signers produced a significantly higher error rate (24.3%; *M* = 3.88 errors, *SD* = 3.41) than either sign learners (14.3%, *M* = 2.28 errors, *SD* = 2.3) or fluent signers (6.3%, *M* = 1.0 errors, *SD* = 1.68) on lateral (ipsilateral-contralateral or vice versa) movements [*X*^2^_(2)_ = 17.23, *p* < 0.001], see Figure 4. Non-signers also produced a significantly higher error rate (2.81%, *M* = 0.45 errors, *SD* = 0.88) than either sign learners (0%) or fluent signers (0%) on inward–outward movements [*X*^2^_(2)_ = 10.20, *p* < 0.01]. There were no group differences in error rates on up–down movements; the non-signers produced two total errors on this parameter, while the sign learners and fluent signers did not produce any errors on this parameter.

##### Palm orientation items

**Figure 5:**
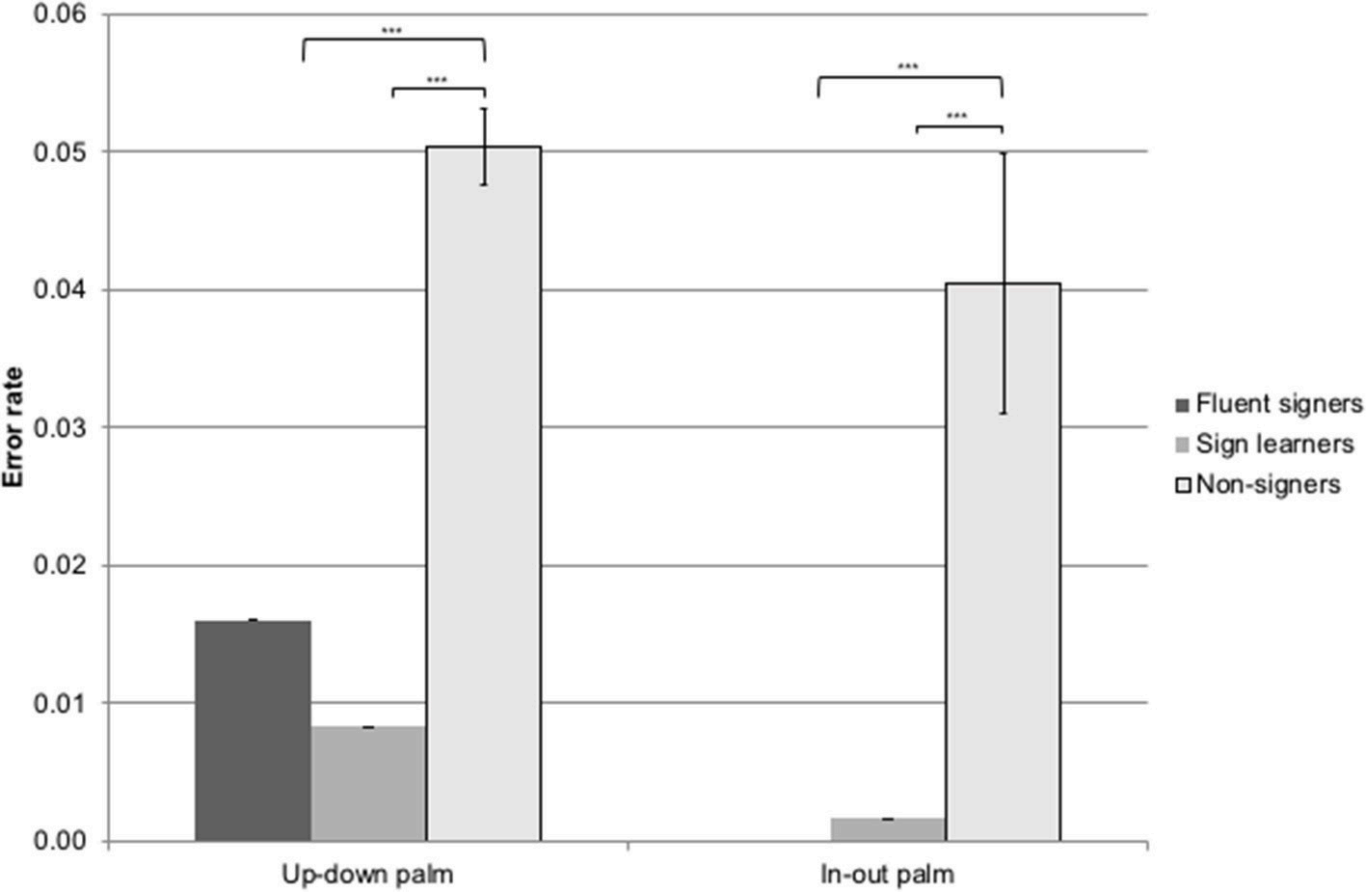
Error rate by palm orientation type for non-signers, sign learners, and fluent signers. ^***^p < 0.001.

Non-signers made more palm orientation errors than either sign learners or fluent signers for both up–down [*X*^2^_(2)_ = 11.80, *p* < 0.01] and in-out [*X*^2^_(2)_ = 20.43, *p* < 0.0001] palm orientation types. Non-signers produced an error rate of 5.04% on up–down palm orientations (*M* = 1.2 errors, *SD* = 1.1), compared to 0.83% (*M* = 0.2 errors, *SD* = 0.1) for sign learners and 1.16% (*M* = 0.28 errors, *SD* = 0.57) for fluent signers. On in-out palm orientations, non-signers produced an error rate of 4.04% (*M* = 0.97 errors, *SD* = 1.58) compared to 0.17% (*M* = 0.04 errors, *SD* = 0.2) for sign learners and 0.23% (*M* = 0.06 errors, *SD* = 0.24) for fluent signers; see Figure 5.

#### Discussion

We predicted that subjects would make more imitation errors on gestures involving lateral movements across the body than on gestures involving vertical or horizontal movements due to a bias toward a mirroring strategy rather than a reversing strategy. Our prediction was borne out: lateral movements were significantly more susceptible to error than other movement types. We further predicted that imitation performance would interact with exposure to sign language, with fluent signers making the fewest number of errors, followed by sign learners, and finally by non-signers (though note that we did not detect statistical differences between the sign learners and the fluent signers). This prediction was also borne out both for the movement and palm orientation gesture types. In particular, non-signers produced a significantly higher rate of reversals on lateral movement gestures (24%) than sign learners (14%) or fluent signers (6%). Non-signers also produced more errors on horizontal (in-out) movements than either sign learners or fluent signers. Importantly, neither signers nor non-signers made errors on the control condition of imitating up–down (vertical) movements.

Non-signers also produced more errors on both kinds of palm orientations than either sign learners or fluent signers. We did not predict these error types; one plausible explanation for their occurrence is that non-signers may have been paying particular attention to the more perceptually salient movements and were paying insufficient attention to palm orientation. Subjects with sign exposure know to pay attention to both movement and palm orientation, since both have linguistic value in sign.

Study 1 showed that certain types of gesture found in signed languages are more difficult to imitate, especially for non-signers who tend to employ the mirroring strategy, leading to lateral movement errors. However, the reversing strategy is only necessary when imitating lateral movements produced by people with the same hand dominance, i.e., right-handers imitating right-handers or left-handers imitating left-handers. Would right-handed non-signers still make more errors on lateral movements if they were imitating a left-handed model, and thus could use a mirroring imitation strategy? In order to test this specific hypothesis, we designed an additional study to examine the role that handedness plays in perspective-taking.

### Study 2: mirroring and discordant handedness

If the difficulty of the reversing strategy is truly at issue in the imitation of lateral movement gestures, then right-handed subjects should only have difficulty imitating other right-handers. Thus, we predicted that right-handed subjects would not have a problem imitating lateral gestures produced by a left-handed model, since such movements can be imitated with a mirroring strategy rather than a reversing strategy.

#### Methods

To test the hypothesis that discordant handedness allows imitators to avoid the reversing strategy on difficult lateral movements, we modified the stimuli used in Study 1.

##### Stimuli

**Figure 6:**
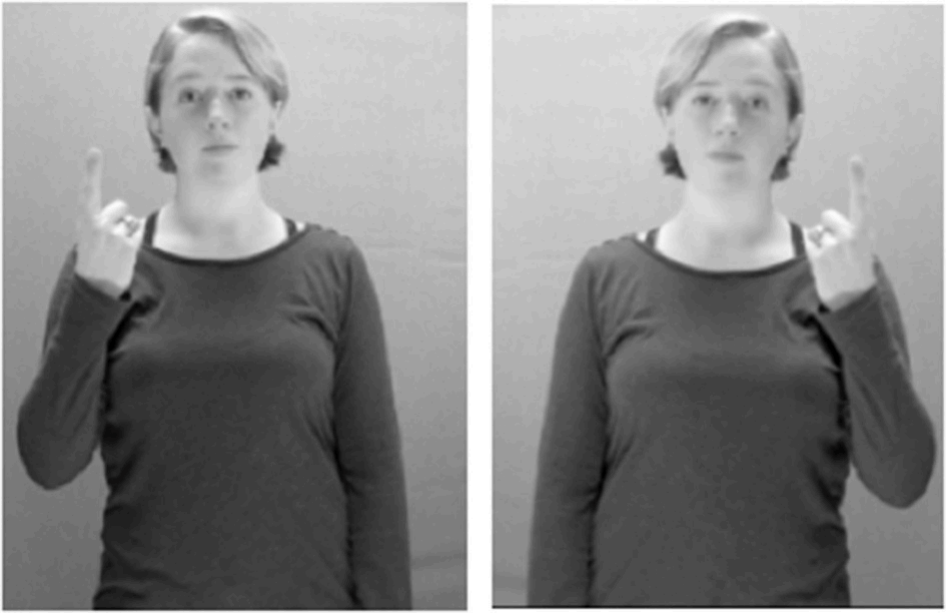
An example of how a gesture stimulus from Study 1 **(left)** was flipped horizontally to appear as if produced by a left-handed gesture model in Study 2 **(right)**. Still images of gesture stimuli are reproduced here and in Table 2 with written permission of the model.

The 48 gesture stimuli used in Study 1 were flipped horizontally such that it now appeared that the right-handed gesture model was producing the gestures with her left hand; see Figure 6. We predicted that right-handed non-signers would not make lateral movement errors in this condition, since they should be able to use mirroring to correctly imitate.

##### Subjects

For Study 2 we recruited 67 non-signing undergraduate students; 34 right-handed non-signers (19 women) were assigned at random to the flipped condition, and 33 right-handed non-signers (27 women) were assigned at random to the same non-flipped condition as in Study 1.

#### Results

**Figure 7:**
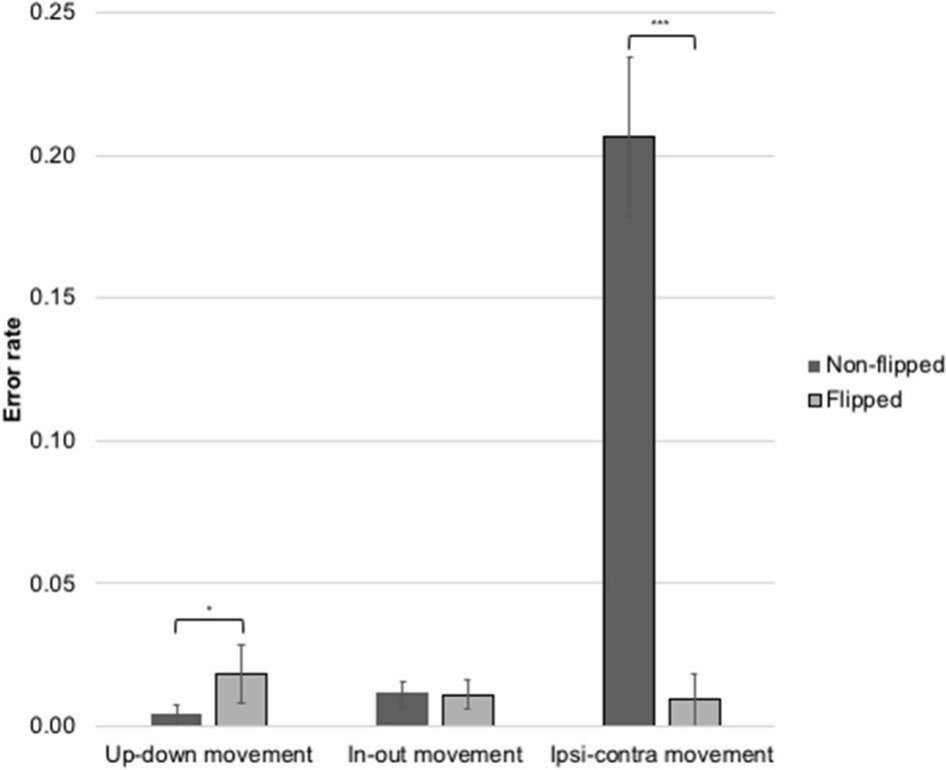
Error rates on movement types in the flipped and non-flipped conditions. Subjects were sign-naive undergraduates. ^***^p < 0.001, ^*^p = 0.05.

Results for Study 2 are shown in Figure 7. In the flipped condition, participants made 10 errors on vertical movements out of 544 trials (1.8%), while in the non-flipped condition, participants made 2 errors on vertical movements out of 528 trials (0.4%). The difference between conditions for vertical movements was marginally significant (Fisher's Exact Test, *p* = 0.05). On horizontal movements, participants in the flipped condition made 6 errors (1.1% of 544 trials); participants in the non-flipped condition also made 6 errors on horizontal movements (1.1% of 528 trials). There was no difference between the two conditions for horizontal movements (Fisher's Exact Test, *p* = 1.0, ns). On lateral movements, participants in the flipped condition made just 5 errors (0.9% of 544 trials), but 109 errors in the non-flipped condition (20.6% of 528 trials). A two-sample Cramer Von-Mises test found that error rate on lateral movements was significantly lower (*p* < 0.001) in the flipped condition than in the non-flipped condition.

No differences were detected between the error rates for palm orientations. Participants produced errors on 1.64% of up–down palm orientations in the non-flipped condition and 2.82% in the flipped condition (Fisher's Exact Test, ns), and 3.41% of in-out palm orientations in the non-flipped condition and 1.47% in the flipped condition (Fisher's Exact Test, ns).

#### Discussion

Study 2 showed that handedness interacts with imitation in specific and predictable ways. First, subjects in the non-flipped condition exhibited a high error rate (20.6%) when imitating lateral movements, replicating the results of Study 1 and confirming that imitating these gestures is difficult. Second, subjects in the flipped condition (who thus appeared to be imitating a left-handed model) made significantly fewer errors (0.9% error rate). We thus demonstrate that gestures exhibiting lateral path movements can be successfully imitated by right-handed subjects when the model being imitated performs the movements with her left hand, thereby enabling a *mirroring* strategy rather than a *reversing* strategy. Thus, we find strong evidence that gesture imitation strategies are influenced by handedness and that lateral movements are easier for non-signers to imitate when handedness is discordant, in line with our predictions. We find no difference in the flipped and non-flipped conditions for palm orientation, in accordance with our prediction that palm orientation would not be affected by the handedness of the model.

The two new gesture imitation studies described here support three hypotheses about the difficulties involved in learning a sign language. First, sign language exposure changes how adults approach imitation, shifting them from a mirroring strategy to a more difficult reversing strategy. Second, lateral movements across the body are more difficult to imitate than either horizontal (inward–outward) or vertical (up–down) movements, since they require a reversing strategy in order to be successfully imitated, provided that the handedness of the imitator and the model is concordant. Since left dominance is relatively rare, concordant handedness is likely to be true of the large majority of sign learning encounters. Third, we demonstrate the role that handedness plays in the imitation of lateral movements, as right-handed non-signers were significantly better at imitating lateral movements when imitating an apparently left-handed model.

## General discussion

We have described some of the ways in which language acquisition in the visual-gestural modality poses unique challenges for sign language learners. Our argument can be summarized as follows:

1. Signed languages use space for several purposes. They can use space to talk about space, as in the descriptions of spatial arrays discussed by Emmorey (2002). They can use space to mark grammatical relations, as in verb agreement and anaphora. Finally, they can use space in a fixed way, as in lexical signs. The sign-learning child must learn to distinguish these different constructions and uses of space. Spatial arrays and grammatical uses of spatial anaphora are relatively advanced skills that appear later in development, but the acquisition of lexical signs occurs early. Children must figure out that lexical signs are not specified for right and left (unlike the spatial layout depicted in Figure 2) but, instead for the movements of the dominant and non-dominant hands.

2. The sign lexicon is composed of different types of signs. Some are one-handed and some are two-handed; two-handed signs may be symmetrical (with both hands exhibiting the same handshapes and movements) or asymmetrical (with each hand exhibiting a different handshape and movement). Signers vary in hand dominance, thus input to children is varied in terms of how they see one-handed and two-handed asymmetrical signs being produced. Children also view signs from various perspectives, further complicating the input they receive.

3. At least four imitation strategies are available for imitating signs. One strategy is the anatomical imitation strategy, in which subjects activate the same muscles as the model they are imitating, resulting in the switching of the hands from dominant to non-dominant when signer and model do not share handedness. We find evidence that typical adults, as well as typical and atypical children, sometimes use this strategy, particularly when imitating difficult gestures. A second strategy is the mirroring strategy, in which subjects produce a mirror image of the gestures or signs they are imitating. We find evidence that typical adults learning sign and imitating gestures tend to use this strategy, and that this results in lateral movement errors when handedness is shared. A third strategy is the visual matching strategy, in which subjects imitate what they see from their own perspective. This leads to reversals in inward–outward palm orientations and inward–outward movements in gesture imitation and sign production. We find evidence that typical adults learning sign, very young typical children, and older hearing and deaf children with ASD sometimes employ this strategy. Finally, skilled signers employ a reversing strategy, in which they perform a mental spatial transformation in order to reproduce the model's gesture. We find that fluent sign language users and sign language learners are better at imitating gestures using the reversing strategy than are non-signers, who prefer the mirroring strategy. We thus find evidence that sign language exposure changes the way that typical adults imitate gestures.

## Conclusion and future directions

We argue that the visual-gestural modality presents challenges to sign language learners unlike the challenges faced by learners of spoken languages. Learners confront variation in input due to differences in handedness in the population, with no obvious analog in speech. One potential analog in speech is the acoustic variation in phoneme production caused by differently-sized vocal tracts, but it is unclear how comparable these two phenomena are. Furthermore the visual-gestural modality allows for multiple ways to interpret the imitation task, while the vocal-auditory modality generally does not. An exception in speech arises in the imitation of pronouns, such that an imitation of the sentence “Mommy loves you” can retain the modeled pronoun or can replace it with “me”, thereby preserving the reference of the model sentence.

In the future we need further work on the acquisition of the sign lexicon by typical deaf children. In particular we need better documentation of their early sign development with regard to the specific predictions made here, especially with respect to the movement and palm orientation types discussed. It would also be interesting to know if signs hypothesized to be difficult to imitate are acquired relatively late in development. A systematic analysis of the MacArthur-CDI database for ASL signs (Anderson and Reilly, 2002; http://wordbank.stanford.edu) could shed light on this problem.

The reports of reversed inward–outward palm orientations in children with ASD, whether hearing children imitating gestures or deaf children producing signs, are a robust indicator that some children with ASD use the visual matching strategy in imitation. However, we still do not have a clear understanding of which children with ASD tend to use this strategy nor how frequently the phenomenon occurs. It may just be a subset of children with ASD who use this strategy rather than being a characteristic strategy of all children with ASD; a crucial question to ask is if those children who employ the visual matching strategy also share a cognitive profile and if other related cognitive characteristics can be identified.

Lastly, we need further work on the gesture development of hearing children in the first 2 years of life. We need systematic documentation of whether or not typical infants reverse the direction of their palm when producing early gestures such as the “wave bye-bye” gesture, when they produce the gesture in its mature form, and what other cognitive milestones occur contemporaneously. Such work on typical deaf and hearing children will put us in a better position to understand the development of sign and gestures in children with ASD. It will also help clarify how children approach imitation and if emergent imitation strategies can be more clearly linked to sign language development.

## Ethics statement

All subjects gave written informed consent in accordance with the Declaration of Helsinki. The protocol was approved by the Institutional Review Board of Boston University.

## Author contributions

AS designed and conducted the original studies reported in this paper, contributed to the development and refining of the hypotheses described in the paper, and was the primary author of the paper. RM contributed to the development and refining of the hypotheses described in the paper, the designing of the original studies reported, and the writing of the paper.

### Conflict of interest statement

The authors declare that the research was conducted in the absence of any commercial or financial relationships that could be construed as a potential conflict of interest.

## Footnotes

^1^In this paper we refer to signs from American Sign Language (ASL). However, the hypotheses advanced here apply to other sign languages. As is conventional in the literature, words in small caps indicate signs.

^2^Notable exceptions in many signed languages are signs indicating cardinal directions (east is typically produced with a rightward direction and west with a leftward direction, in correspondence with the directions on a compass); the same is true for the lexical signs right and left. We thank one of the two reviewers for this observation.

^3^An imperfect analogy is perhaps the different vocal tract sizes and resulting fundamental frequencies of male and female speakers (Peterson and Barney, 1952). Six-month-old hearing infants are able to recognize phonemes produced by speakers despite great acoustic variation (*talker normalization*; Kuhl, 1979).

^4^We do not suggest that imitation is the only mechanism through which children acquire linguistic symbols. We focus here on imitation as one essential component of the language-learning process.

^5^Note that a right-dominant signer can correctly imitate a left-handed model (or a left-dominant signer, a right-handed model) using the mirroring strategy. Under this scenario, a signer would monitor the hand dominance of the model and then employ mirroring if handedness is discordant.

^6^Although we did not collect data on the ages of participants, it is worth noting that the fluent signers were working adults or graduate students, while the other two groups were undergraduate students.
